# Protein-Losing Enteropathy in Crohn's Disease: Two Unusual Cases

**DOI:** 10.7759/cureus.19501

**Published:** 2021-11-12

**Authors:** Samira Samant, Danielle R Lyon, Nisar Asmi, Pinky Jha

**Affiliations:** 1 Internal Medicine, Medical College of Wisconsin, Wauwatosa, USA

**Keywords:** low serum albumin, alpha-1 antitrypsin, irritable bowel disease, crohn’s disease (cd), protein-losing enteropathy

## Abstract

Protein-losing enteropathy (PLE) occurs when protein losses throughout the gastrointestinal (GI) tract exceed the liver’s ability to produce new protein. This is a rare presentation of Crohn's disease and few reported cases of PLE related to Crohn’s exist in the literature. We describe two patients who presented with symptoms of PLE. After extensive diagnostic workup both were diagnosed with Crohn’s disease and managed with prednisone.

## Introduction

Hypoalbuminemia has a variety of underlying etiologies. These fall into the categorization of either decreased production or increased protein loss. Protein loss may occur through the kidney, GI tract, in the context of severe burns or sepsis, or via third-spacing of fluid [1]. In a patient without overt signs or symptoms corresponding to one of these mechanisms, identifying the underlying mechanism may be challenging. Therefore, broad differentials must be maintained. 

Protein-losing enteropathy (PLE) occurs when protein losses throughout the GI tract exceed the liver’s ability to synthesize novel proteins [2]. Underlying mechanisms include increased lymphatic pressure (lymphangiectasias), gastrointestinal (GI) diseases with mucosal erosions and inflammation, or GI pathology without mucosal erosion (such as celiac disease) [2].

## Case presentation

Case 1

A 53-year-old woman with a history of deep vein thrombosis (DVT), hypothyroidism, and small bowel obstruction with partial resection presented with a one-month history of watery diarrhea with abdominal distension, weight gain, and progressively worsening edema of the upper and lower extremities. Her blood pressure was 88/51 at presentation, but she noted that her systolic pressures were usually in the 90s. The patient denied fever, vomiting, headache, and dysuria. Physical exam was notable for distended abdomen with diffuse tenderness to palpation. Bowel sounds were normoactive and no hepatosplenomegaly was noted. In addition, the patient had non-pitting edema involving all four extremities, temporal wasting, and anasarca.

**Figure 1:**
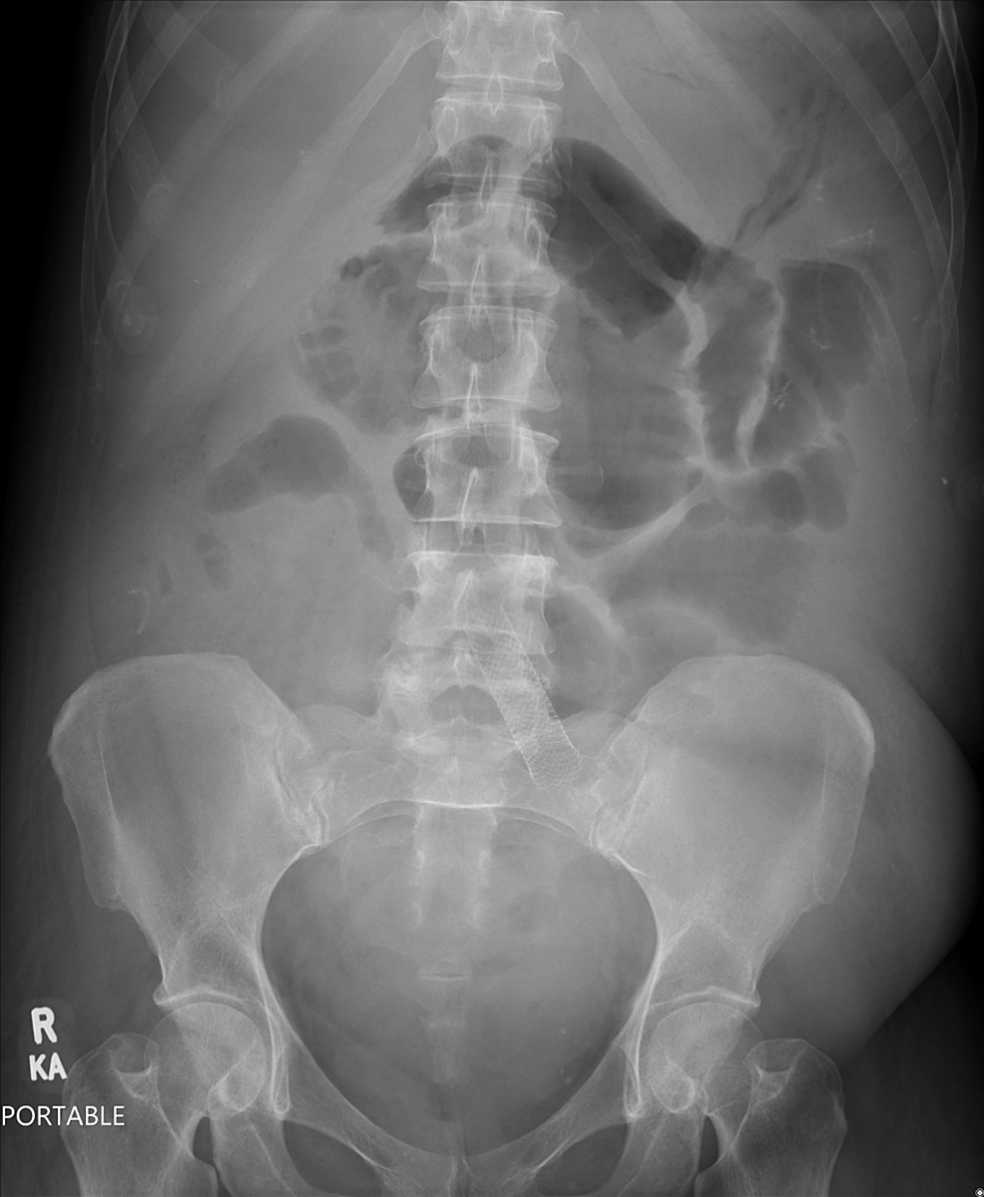
KUB demonstrating gaseous dilation of the small bowel, consistent with mild ileus. KUB, kidney, ureter, and bladder

Laboratory workup upon admission was significant for hypoalbuminemia with an albumin level of 1.9 mg/dL and total protein of 3.1 mg/dL. Potential etiologies of the low protein were considered including nephrotic syndrome, liver, or cardiac disease. Urinalysis was negative for protein, ruling out nephrotic syndrome. Similarly, liver disease was ruled out due to normal liver enzymes and cardiac disease was unlikely given normal function on echocardiogram. Celiac antibody, *Clostridium difficile*, and stool culture tests were all negative. Further workup included an abdominal CT which was significant for proximal and distal small bowel inflammation and wall thickening, but the findings were not classic for Crohn's disease. In addition, the patient did not have the overt symptoms of Crohn's that would be suspected given the degree of bowel involvement. Echocardiographic findings were insignificant, and abdominal X-ray showed mild ileus of the small bowel (Figure 1).

**Figure 2:**
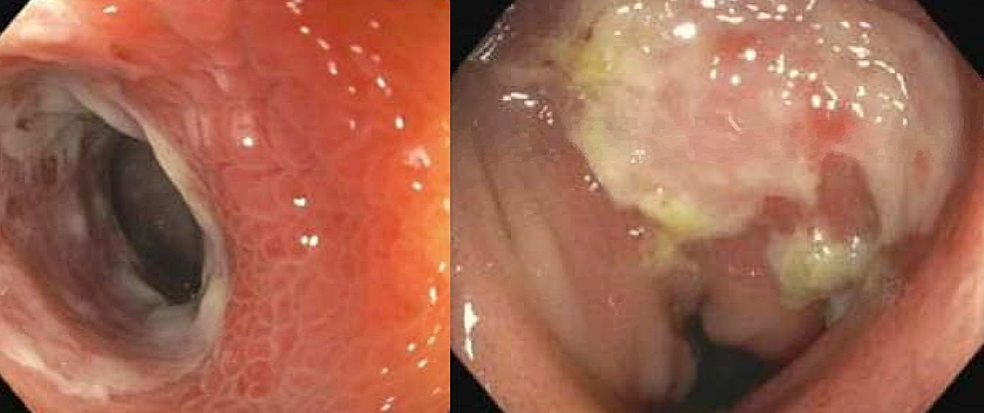
Colonoscopy images showing erythema and TI ulceration (left), and a second TI ulcer (right). TI, terminal ileum

Ultrasound of her extremities noted bilateral lower extremity DVTs and DVT of the right upper extremity. Chest CT ruled out pulmonary embolism. Upper endoscopy revealed a mildly dilated duodenal bulb. Biopsies of both the stomach and duodenal bulb were negative for *Helicobacter pylori*. Colonoscopy was significant for ulcerated and erythematous ileal mucosa interspersed with normal appearing mucosa (Figure 2).

Biopsy results confirmed active ileitis with ulceration, consistent with Crohn’s disease. Alpha-1-antitrypsin stool collection confirmed the diagnosis of PLE due to Crohn’s. The patient was started on IV methylprednisolone and monitored with good response. Due to her continued improvement, she was switched to oral prednisone and discharged the next day. At two week follow-up, the patient began steroid taper with plans to start infliximab infusions and monitor her response to therapy as an outpatient.

Case 2

**Figure 3:**
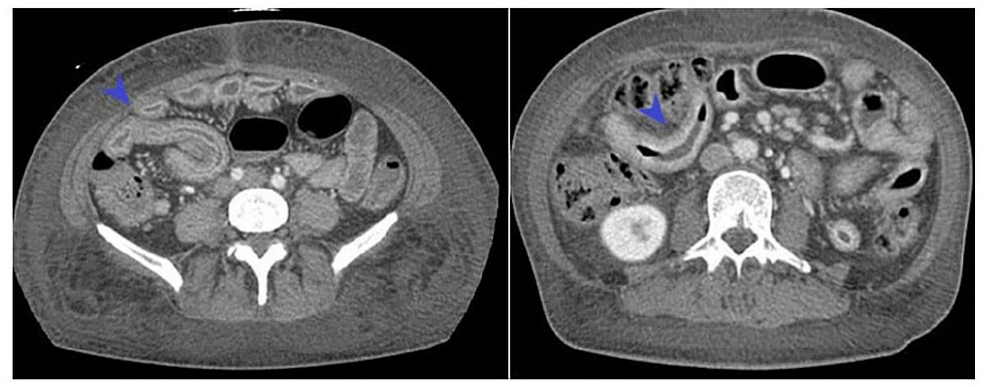
CT abdomen and pelvis, demonstrating diffuse small bowel wall thickening from the distal jejunum to TI, along with mesenteric adenopathy. TI, terminal ileum

A 47-year-old man with history of diabetes mellitus presented with one month history of watery diarrhea with diffuse crampy abdominal pain, progressive abdominal distension, weight loss, and progressively worsening bilateral lower extremity edema. The patient denied fever, nausea, vomiting, tenesmus, and hematochezia. Physical exam was notable for diffuse tenderness to palpation and generalized anasarca. Laboratory workup was significant for hypoalbuminemia with an albumin level of 1.9 mg/dL. HIV, stool cultures, parasitology, and cryptosporidium were repeatedly negative. CT abdomen and pelvis showed diffuse bowel thickening from the distal jejunum to the terminal ileum with mesenteric adenopathy (Figure 3).

**Figure 4:**
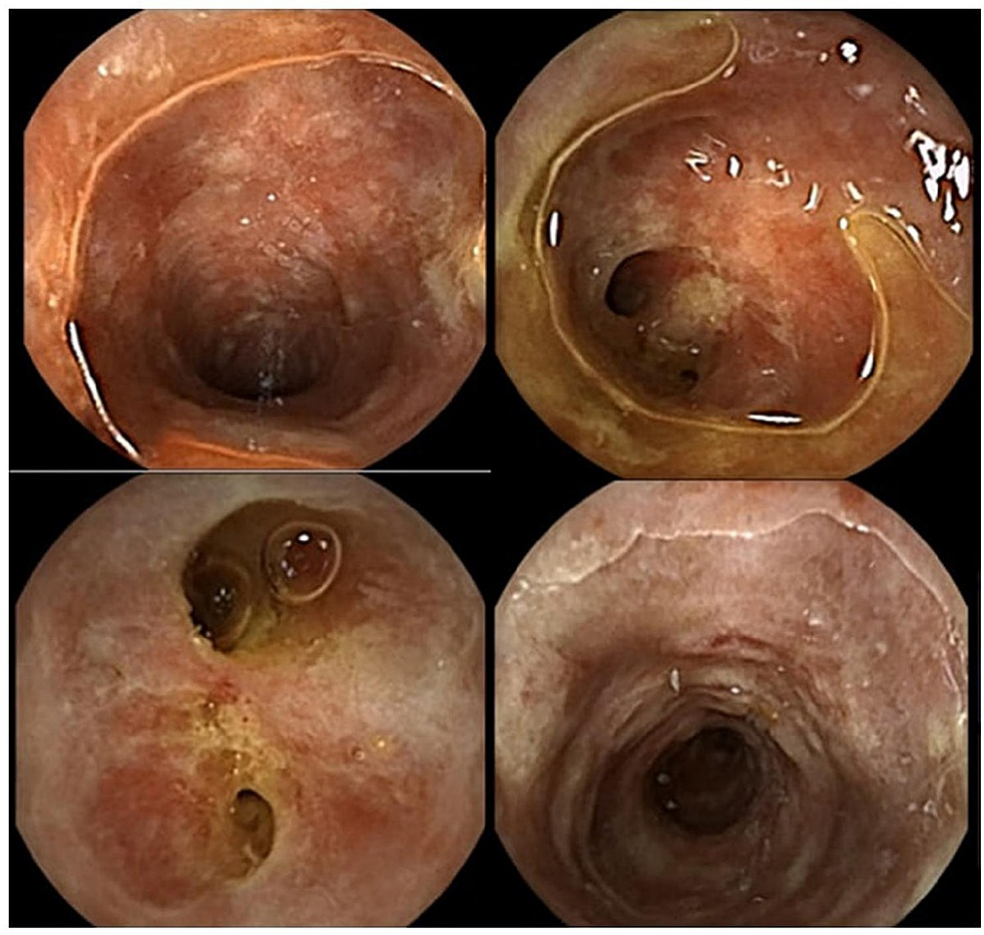
Video capsule endoscopy showing multiple small bowel ulcers with skip lesions and possible strictures.

Upper endoscopy and colonoscopy revealed few gastric erosions with mild edematous colonic mucosa and no evidence of disease in the terminal ileum (TI). Biopsies were negative for viral, fungal, and acid-fast bacilli (AFB) cultures, and the patient was discharged with instructions for close follow-up. Post-discharge tests came back with a positive fecal fat and anti-Saccharomyces cerevisiae antibodies. The patient returned three months later with similar complaints and capsule endoscopy was performed due to lack of significant findings in previous endoscopies. This showed multiple small bowel ulcers covering 63% of the length of the small bowel with skip areas and possible strictures, consistent with Crohn’s disease (Figure 4).

**Figure 5:**
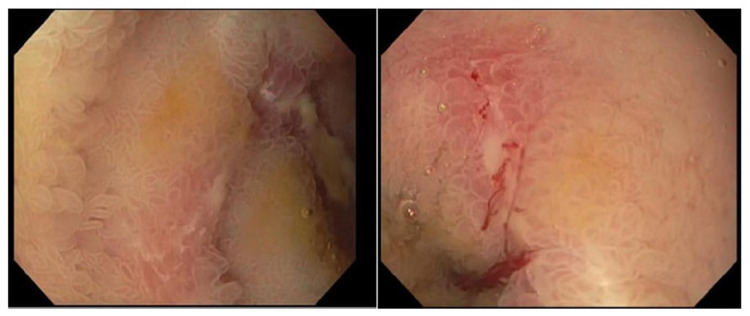
Retrograde single-balloon enteroscopy demonstrating multiple ulcers in the ileum.

Retrograde single-balloon enteroscopy was performed and showed multiple medium to large ulcers in the ileum. The ulcers started 20-25 cm proximal to the ileocecal valve (Figure 5).

Multiple biopsies showed mixed inflammatory infiltrate. Immunohistochemistry for lymphoma, special stains for fungal infection, and AFB were all negative. The patient was discharged on prednisone with improvement at his follow-up visit. 

## Discussion

The PLE related to Crohn’s disease is a rare disorder; in our review of the current literature, we identified 13 reported cases. To our knowledge, no incidence or prevalence has been reported for PLE in Crohn’s, likely due to the under-reported and under-diagnosed nature of this condition. We aim to publish this case to increase awareness among clinicians to consider this diagnosis earlier in the disease course, as retaining it in the differential may prevent significant morbidity and mortality.

The diagnosis is made by measuring the clearance of alpha-1 antitrypsin -- calculated by the ratio of 24-h stool quantity to serum levels [3]. The gold standard for diagnosing PLE is measuring fecal excretion of 51Cr-labeled albumin. However, this test is complex and difficult to perform, making fecal alpha-1 antitrypsin the preferred method in most clinical settings [2-3].

Presentations of PLE vary greatly based on the underlying etiology, which can make diagnosis challenging. Edema and hypoalbuminemia on laboratory workups are common preliminary findings. Other less frequent signs and symptoms include anasarca, pleural or pericardial effusions, and macular edema [3]. In both cases reported here, the patient presented with generalized edema, abdominal distension, anasarca, and watery diarrhea. Given their GI symptoms in the context of hypoalbuminemia identified in laboratory studies, our workup centered around evaluating for an underlying GI pathology. With negative celiac and infectious workups in both cases, we identified Crohn’s disease via concerning CT findings that ultimately led to diagnostic colonoscopies. Also valuable in the evaluation for inflammatory bowel diseases are inflammatory serologic or fecal markers. C-reactive protein, a serologic marker of inflammation, has been demonstrated to have greater specificity for Crohn's disease than ulcerative colitis; it has a short half-life and responds rapidly to changes in the severity of systemic inflammation [4]. Fecal calprotectin, a fecal biomarker, strongly correlates with indium-111-labeled granulocyte scintigraphy, the gold-standard method for detecting inflammation in inflammatory bowel disease (IBD); it is useful both in detecting systemic inflammation prior to the onset of clinical symptoms and in disease monitoring, although in the latter it is more predictive of relapse in ulcerative colitis than Crohn's [4]. These and other biomarkers, including lactoferrin and α-1 antitrypsin, provide useful noninvasive methods of assessing inflammation and monitoring IBD in affected patients.

The underlying mechanism of PLE in Crohn’s disease is likely due to the development of mucosal erosions and ulcerations. These permit protein loss through inflammatory exudation and absorptive loss across the eroded epithelial barrier [5]. It has been suggested in the literature that there may also be a component of increased lymphatic pressure contributing to PLE in Crohn’s disease [2, 6]. This theory, based upon the known maintenance of lymphatic vessels in inflammatory settings, suggests that lymphatic and blood vessel interactions may play a role in the presentation of inflammatory bowel diseases. It is supported by early reports of Crohn’s disease and ulcerative colitis describing colonic lymphatic congestion and remodeling [6]. This being said, the local lymphangiogenesis, lymphatic obstruction, and submucosal edema seen in Crohn’s disease are not expected to significantly elevate lymphatic pressure. However, central blockages of systemic lymphatic channels elevate lymphatic pressure by creating ruptures through which total body lymph leaks into the GI tract [2]. While there may be some contribution of lymphatic pressure to the development of PLE in Crohn’s, mucosal breakdown caused by chronic, localized inflammation remains the primary mechanism.

Notably, the degree of mucosal involvement in inflammatory bowel disease often correlates with the severity of protein loss. This relationship allows protein loss through the GI tract to be utilized as a marker for the severity of the underlying disease itself. Fecal alpha-1 antitrypsin clearance is the test of choice for both diagnosing PLE and assessing IBD severity [7].

Ultimately, the diagnosis of PLE in Crohn’s disease is challenging, given the lack of symptom specificity at initial presentation and the complexity of the resulting workup. Comprehensive initial lab workup is crucial, as it identifies hypoalbuminemia while also evaluating for the presence of renal or hepatic abnormalities. In the absence of these, along with a low suspicion for malnutrition and a history of diarrhea in both patients, we focused on a GI-related etiology. While infectious causes of GI illness and celiac disease must be ruled out, obtaining imaging to evaluate for the presence of IBD in such cases is vital. Without this, the diagnosis is extremely difficult to make.

Identification of the underlying disease is crucial in PLE, as treatment is centered around addressing the root cause. PLE is less a disease in and of itself, but more a complicating development in the course of another disease process [3]. In the case of Crohn’s disease, there is evidence that PLE may alter the typical seven- to ten-day half-life of infliximab, and that GI protein loss may play a role in the more rapid metabolism of anti-TNF globulins that has been demonstrated in active Crohn’s [2, 8]. Apart from such interactions, where the therapeutic course must be closely monitored, PLE typically responds well to treatment of the underlying disease [5]. While working up the underlying etiology often proves challenging, and a great deal of research remains to be done in this field to promote early diagnosis and treatment, doing so will make a great deal of difference in both ameliorating the patient’s symptoms and preventing further complications.

## Conclusions

Protein-losing enteropathy in Crohn's disease is difficult to recognize and therefore historically under-diagnosed. Consequently, a diagnostic framework is challenging given the variety of manifestations that may be seen. By presenting this case we hope to contribute to the growing body of literature regarding the development of PLE in Crohn's disease. The development of PLE often heralds a worsening severity of the underlying disease and early recognition is important. We aim to present our clinical reasoning and diagnostic workup to illustrate how we arrived at a diagnosis of Crohn's given a presentation of PLE.
